# Immune patterns of cuproptosis in ischemic heart failure: A transcriptome analysis

**DOI:** 10.1111/jcmm.18187

**Published:** 2024-03-20

**Authors:** Zhebin Chen, Yunhui Zhu, Songzan Chen, Zhengwei Li, Guosheng Fu, Yao Wang

**Affiliations:** ^1^ Department of Cardiology, Sir Run Run Shaw Hospital School of Medicine, Zhejiang University Hangzhou People's Republic of China; ^2^ Key Laboratory of Cardiovascular Intervention and Regenerative Medicine of Zhejiang Province Hangzhou People's Republic of China

**Keywords:** cuproptosis, immune microenvironment, immunity, ischemic heart failure, transcriptome analysis

## Abstract

Cuproptosis is a recently discovered programmed cell death pattern that affects the tricarboxylic acid (TCA) cycle by disrupting the lipoylation of pyruvate dehydrogenase (PDH) complex components. However, the role of cuproptosis in the progression of ischemic heart failure (IHF) has not been investigated. In this study, we investigated the expression of 10 cuproptosis‐related genes in samples from both healthy individuals and those with IHF. Utilizing these differential gene expressions, we developed a risk prediction model that effectively distinguished healthy and IHF samples. Furthermore, we conducted a comprehensive evaluation of the association between cuproptosis and the immune microenvironment in IHF, encompassing infiltrated immunocytes, immune reaction gene‐sets and human leukocyte antigen (HLA) genes. Moreover, we identified two different cuproptosis‐mediated expression patterns in IHF and explored the immune characteristics associated with each pattern. In conclusion, this study elucidates the significant influence of cuproptosis on the immune microenvironment in ischemic heart failure (IHF), providing valuable insights for future mechanistic research exploring the association between cuproptosis and IHF.

## 1 INTRODUCTION

Heart failure (HF) is the end stage of almost all cardiovascular diseases (CVDs) and is characterized by impaired ventricular blood filling or ejection.^1^ The leading causes of HF include ischemic heart diseases such as myocardial infarction (MI) and atherosclerosis, valvular heart diseases (VHD), hypertension and cardiomyopathy.^1^ Among these, ischemic heart diseases play a crucial role in both acute and chronic heart failure.^2^ In clinical practice, left ventricular ejection fraction (LVEF) and N‐terminal pro‐brain natriuretic peptide (NT‐proBNP) serve as commonly empolyed indicators for screening HF and assessing severity.^3^ Despite decades of extensive research aimed at identifying novel biomarkers for predicting and stratifying HF risk,^4^, ^5^, ^6^, ^7^ a substantial number of HF cases occur in individuals devoid of conventional risk factors such as age, sex, hypertension, diabetes and smoking.^8^ Recent clinical studies have sought to establish more comprehensive risk assessment tools for HF, yet the current methodologies remain insufficient.^9^ Hence, there exists an urgent demand for more accurate and comprehensive risk assessment tools that encompass factors like genetic predisposition, biomarker levels and lifestyle choices. Further research is imperative to uncover new risk factors and enhance current risk models, thereby bolstering our capacity to predict and prevent HF. The advent of next‐generation sequencing (NGS) has contributed to the increasing popularity of genetic prediction models based on gene sequencing results in both clinical practice and scientific research. However, reports on predicting HF through genetic expression remain scarce. Expanding our understanding in this area could provide valuable insights for improved HF prediction and management strategies.

Copper (Cu) is a vital micronutrient essential for maintaining human physiological homeostasis and influencing pathological processes.^10^ Elevated serum copper levels have been observed in patients with HF, suggesting a potential association between copper and HF.^11^ However, further validation through fundamental laboratory experiments is required. Recently, researchers discovered a new form of programmed cell death triggered by excessive intracellular copper levels, named cuproptosis.^12^ Cuproptosis involves copper binding to lipoylated enzymes in the tricarboxylic acid (TCA) cycle, leading to the aggregation of lipoylated proteins, proteotoxic stress and ultimately cell death.^12^ Protein lipoylation, especially lipoic acid (LA)‐mediated lipoylation of pyruvate dehydrogenase (PDH) complex components, plays a crucial role in cuproptosis. Key components of the LA pathway include FDX1, LIPT1, LIAS and DLD, while the PDH complex primarily consists of DLAT, PDHA1 and PDHB.^13^ The regulation of protein lipoylation by FDX1 is a crucial step in initiating cuproptosis. Similar to other programmed cell death patterns such as ferroptosis and pyroptosis,^14^, ^15^ cuproptosis is likely to significantly contribute to the development and progression of CVDs, considering the importance of cell death and remodelling in these conditions.^16^ Recent studies have explored the molecular mechanisms associated with cuproptosis in diabetic cardiomyopathy^17^ and ischemic stroke.^18^ HF is tightly associated with mitochondrial dysfunction and energy metabolism defect,^19^ and HF is also believed to involve both cell death and remodelling in pathological changes.^20^ Due to the critical role of the TCA cycle in mitochondrial function and energy metabolism, cuproptosis is probably involved in the progress of HF, although current research in this area remains unclear.

The immune response and inflammatory reactions in the pathological microenvironment have long been acknowledged as pivotal contributors to the early onset and subsequent remodelling of HF.^21^ In ischemic heart failure (IHF), the substantial infiltration of inflammatory cells, including neutrophils, monocytes and macrophages, into the infarcted region results in the establishment of an early inflammatory microenvironment.^22^ The polarization of infiltrated neutrophils and macrophages subsequently influences the remodelling of the infarcted ventricle.^23^, ^24^ Moreover, dendritic cells (DC) play a significant role in antigen presentation, facilitating adaptive immune responses, particularly the activation of T lymphocytes and B lymphocytes.^25^, ^26^ While in long‐term chronic heart failure, toll‐like receptor 4 (TLR4) has shown to consecutively trigger signalling cascades that promote the activation of NF‐κB, interferon regulatory factor transcription factors and activator proteins, leading to the expression of inflammatory genes across a broad spectrum.^27^ Given the close interconnection between cuproptosis and the TCA cycle, as well as energy metabolism, both of which are intimately associated with immune reactions,^28^ it is worthwhile to explore the relationship between cuproptosis and immune activities in the progress of HF.

In this study, we conducted a systematic analysis of the differential expression of cuproptosis‐related genes between healthy and IHF hearts using samples with sequencing data from Gene Expression Omnibus (GEO) database. Our analysis revealed cuproptosis‐related genes could be applied for distinguish healthy and IHF samples according to the differential expression, suggesting their potential as diagnostic markers. We then developed a HF risk prediction model based on these cuproptosis‐related genes. Additionally, we found a strong association between these genes and the observed immune characteristics in IHF. To further investigate the regulatory role of cuproptosis in IHF, we performed unsupervised clustering and identified two distinct subtypes based on the expression of cuproptosis genes in IHF. These subtypes exhibited specific immune microenvironment characteristics, including immunocyte infiltration, immune response and Human Leukocyte Antigen (HLA) status. Furthermore, we conducted a comparative analysis of the biological functions associated with these subtypes. These findings of our study provide compelling evidence supporting the hypothesis that the expression of cuproptosis genes plays a regulatory role in IHF, particularly through the modulation of the immune microenvironment.

## 2 MATERIALS AND METHODS

### 2.1 Data process

The dataset utilized in this study comprised a total of 231 samples, including 136 samples from healthy individuals and 95 ischemic left ventricles samples from six HF patients and healthy individuals. The sample processing protocol and RNA extraction procedure were described in detail in a previous study.^29^ For gene expression analysis, we used the Affymetrix Human Gene 1.1 ST Array following the manufacturer's instructions. The data for this study was retrieved from the GEO database with the serial number GSE57338 (https://www.ncbi.nlm.nih.gov/geo/query/acc.cgi?acc=GSE57338). The data preprocessing steps employed in this study were consistent with our previous research and are illustrated in Figure S1.^30^

### 2.2 Alteration analysis of cuproptosis related genes between healthy and ischemic heart failure samples

An analysis was conducted to investigate the differences in cuproptosis genes between healthy and IHF samples. The R package ‘limma’ was utilized for the differential analysis, identifying differentially expressed cuproptosis genes with an adjusted *p*‐value <0.01 and |log2FC| >0.5. The protein–protein interaction network was constructed using the STRING database (https://string‐db.org/).^31^ To initially identify cuproptosis genes related to IHF, univariate logistic regression with a *p*‐value cutoff of <0.0001 was performed. Subsequently, a least absolute shrinkage and selection operator (LASSO) regression was employed to select the most informative biomarkers from the pool of 10 cuproptosis genes related to IHF. The R package ‘glmnet’ was used to construct a diagnostic model with non‐zero coefficients. Furthermore, multivariate logistic regression was employed to develop a classifier specifically relevant to cuproptosis genes in IHF. Risk scores were calculated throughout the analysis based on the regression coefficients of cuproptosis genes associated with IHF, representing the individual's risk of developing IHF. The risk score equation is defined as follows:

Risk Score = 88.058 + (−4.295) × MTF1 + (−5.89) × FDX1 + (−5.957) × DLAT +1.065 × LIPT1 + 2.968 × GLS + 3.636 × PDHB + 5.646 × LIAS + 4.906 × DLD + 0.088 × CDKN2A + (−10.184) × PDHA1.

Principal components analysis (PCA) was subsequently applied to reduce the dimensionality of the data and identify similarities and differences in risk scores between the healthy and IHF samples. Additionally, a receiver operating characteristic (ROC) curve was plotted to evaluate the classification performance of the classifier.

To further evaluate the robustness of our model, we utilized sequencing data from three additional databases GSE26887 (5 healthy samples and 12 IHF samples), GSE42955 (5 healthy samples and 12 IHF samples) and GSE76701 (4 healthy samples and 4 IHF samples) for validation.

### 2.3 Correlation analysis between cuproptosis genes and immune characteristics

To investigate the relationship between cuproptosis genes and immune characteristics in IHF, we conducted correlation analysis between cuproptosis genes and infiltrating immunocytes, as well as gene‐sets associated with immune reactions. We evaluated the population of specific infiltrating immunocytes and the activity of immune reactions by employing single‐sample gene set enrichment analysis (ssGSEA). The gene‐sets for infiltrating immunocytes were obtained from a previous study,^32^ while the gene sets for immune reactions were obtained from the ImmPort database (http://www.immport.org).^33^ The Wilcoxon rank‐sum test was employed to calculate the enrichment scores for immunocyte abundance and immune reaction activity.

### 2.4 Identification of cuproptosis expression patterns

To identify the expression patterns of all 10 cuproptosis genes, unsupervised clustering analysis was performed. Consensus clustering algorithm was utilized to evaluate the expression patterns of cuproptosis genes using the R package ‘ConsensuClusterPlus’.^34^ A total of 1000 iterations were performed to ensure the robustness and reliability of the classification results. Additionally, PCA was used to validate the expression patterns of the 10 cuproptosis genes. The Kruskal test was used to compare the infiltrating immunocyte abundance score, immune reaction score and HLA gene expression between the two distinct expression patterns.

### 2.5 Biological enrichment analysis of the two cuproptosis expression patterns

To assess the biological changes related to cuproptosis, we identified the differentially expressed genes (DEGs) between the identified subtypes using the R package ‘limma’.^35^ The criterion for DEGs was |log2FC| >0.5 and adjusted *p*‐value <0.001. Next, we employed the HALLMARKS and KEGG pathways to identify cuproptosis‐related biological signalling pathways. The gene‐sets for ‘h.all.v7.0.symbols’ and ‘c2.cp.kegg.v7.0.symbols’ were downloaded from MSigDB (http://www.gsea‐msigdb.org/).^36^ Gene‐set variation analysis (GSVA) was utilized to calculate the enrichment scores of HALLMARKS and KEGG pathways.^37^ Pathways with an adjusted *p*‐value <0.01 were considered to indicate significant differences between the two subtypes. The biological function of genes related to the cuproptosis phenotype was analysed through GO‐BP and KEGG enrichment analysis using the R package ‘clusterProfiler’.^38^

### 2.6 Identification of cuproptosis expression pattern related gene modules

To identify gene modules related to cuproptosis regulation patterns, we calculated the coefficient of variation (CV) for each gene based on its expression and removed genes with low variability (CV <0.1). Next, we utilized weighted gene co‐expression network analysis (WGCNA) to identify modules of genes that exhibited strong correlation across all samples.^39^

## 3 RESULTS

### 3.1 The landscape of cuproptosis genes between healthy and IHF samples

**FIGURE 1:**
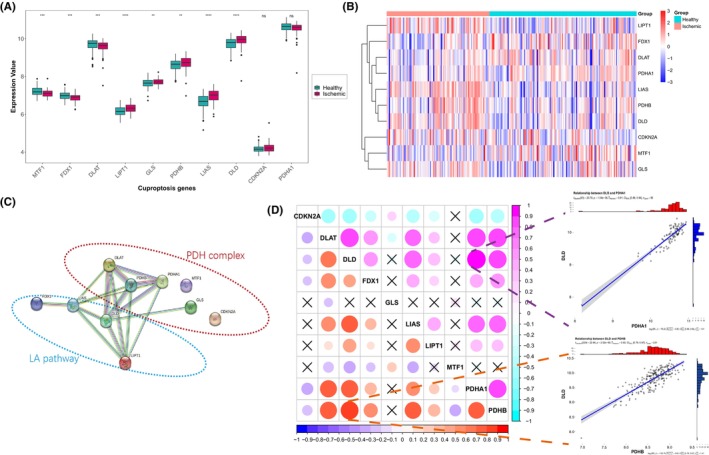
Expression landscape of cuproptosis genes in ischemic heart failure (IHF). (A) The box‐plot demonstrated the transcriptome expression status of 10 cuproptosis genes between healthy and IHF samples. Wilcoxon rank‐sum test was employed for significant testing. (**** < 0.00001, *** < 0.0001, ** < 0.001, * < 0.05, ns–not significant). (B) The heatmap demonstrated the transcriptome expression status of 10 cuproptosis genes between healthy and IHF samples. (C) The composition summary of the protein–protein interactions among 10 cuproptosis genes. (PDH complex: pyruvate dehydrogenase complex; LA pathway: lipoic acid pathway). (D) Correlations among the expression of 10 cuproptosis genes in the IHF samples (Upper triangle) and all samples (Lower triangle). The two scatter‐plots demonstrated the most correlated Cuproptosis genes: DLD and PDHA1 for IHF samples, DLD and PDHB for all samples.

To investigate the expression status in IHF, we selected 10 cuproptosis‐related genes that had been previously reported.^12^ We analysed the RNA‐seq data sampled from 136 healthy and 95 ischemic left ventricles from GSE57338, obtaining the expression levels of these 10 cuproptosis genes. The differential analysis revealed significant expression alterations in eight cuproptosis genes, with three genes showing upregulation and five genes exhibiting downregulation (Figure 1A,B). Notably, the expression of FDX1 was downregulated, whereas the expression of all three LA pathway‐related genes (LIPT1, LIAS and DLD) showed upregulation. Additionally, the PDH complex‐related genes exhibited changes in various directions.

To elucidate the interactions among these cuproptosis genes, we constructed a protein–protein interaction network. As Figure 1C illustrated, FDX1 was found to be regulated upstream by interacting with LIAS, and LIAS interacted with two other LA pathway components, DLD and LIPT1. Furthermore, these three LA pathway proteins interacted with four PDH complex components (DLAT, PDHB, PDHA1 and GLS), among which DLAT, PDHB and PDHA1 displaying tighter interactions. Correlation analysis further revealed a strong relationship between the cuproptosis genes, the LA pathway and the PDH complex. Notably, DLD and PDHB exhibited the highest correlation among all samples (Figure 1D). Whereas in IHF samples, the strongest correlation was observed between DLD and PDHA1 (Figure 1D). Moreover, a primary correlation was observed between the two main components of the LA pathway (LIAS and DLD) and three components of the PDH complex (DLAT, PDHB and PDHA1).

### 3.2 Cuproptosis genes can well distinguish healthy and IHF samples

**FIGURE 2:**
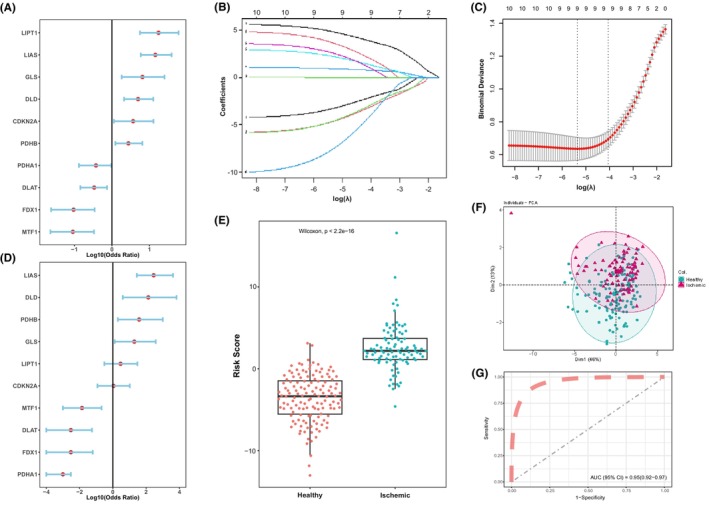
Cuproptosis genes can distinguish healthy and IHF samples. (A) Univariate logistic regression investigated the relationship between 10 cuproptosis genes and IHF (X intercept 0 means *p* < 0.05). (B) Least absolute shrinkage and selection operator (LASSO) coefficient profiles of 10 cuproptosis genes. (C) 10‐fold cross‐validation for tuning parameter selection in the LASSO regression. Partial likelihood deviance values are shown, with error bars representing SE. The dotted vertical lines are drawn at the optimal values by minimum criteria and 1‐SE criteria. (D) Distinguishing signature with 10 cuproptosis genes was developed by multivariate logistic regression, and the risk scores for IHF were calculated (X intercept 0 means *p* < 0.05). (E) The risk distribution between Healthy and Ischemic heart failure group, where Ischemic group has a much higher risk score than healthy samples. Wilcoxon rank‐sum test was employed for significant testing. (F) Principal component analysis (PCA) of 10 cuproptosis genes between Healthy and Ischemic samples. (G) The discrimination ability for healthy and IHF samples by 10 cuproptosis genes was analysed by ROC curve and evaluates by AUC value.

To investigate the contribution of cuproptosis genes to the pathogenesis of IHF, a series of biological algorithms were conducted. Initially, we performed univariate logistic regression to identify cuproptosis genes associated with IHF. Remarkably, all 10 cuproptosis genes exhibited a strong association with IHF (Figure 2A). Subsequently, we utilized LASSO regression for feature selection and dimension reduction, which confirmed the essential role of all 10 genes in the context of IHF (Figure 2B,C). Next, we performed multivariate logistic regression to develop a classifier capable of distinguishing healthy samples from those with IHF and calculated the corresponding risk scores (Figure 2D). We found that this classifier, comprising the 10 cuproptosis genes, exhibited effective discrimination between healthy and IHF samples. The IHF prediction model was constructed using the multivariate regression coefficients of the cuproptosis genes, and risk scores were calculated for each sample. Notably, higher risk scores were observed in IHF sample compared to healthy samples (Figure 2E). Principal Component Analysis (PCA) revealed distinct cuproptosis gene expression patterns between healthy and IHF samples (Figure 2F). Additionally, the Receiver Operating Characteristic (ROC) curve illustrated the strong performance of the cuproptosis IHF prediction model in distinguishing healthy and IHF samples, with an area under the curve (AUC) of 0.95, indicating the robustness of the model (Figure 2G). Furthermore, we performed external validation of our cuproptosis IHF prediction model using data from other databases (GSE26887, GSE42955 and GSE76701). The AUC of the validation ROC curve was 0.72 (Figure S2), confirming the robustness of our model.

### 3.3 Cuproptosis genes are associated with the immune characteristics of IHF

Numerous studies have previously detailed the involvement of the immune system in the pathophysiological mechanisms of IHF. In exploring the link between cuproptosis genes and the immune microenvironment, we conducted a correlation analysis of these genes with both infiltrating immune cells and immune response gene sets, following a methodology paralleling that of an earlier study.^40^

**FIGURE 3:**
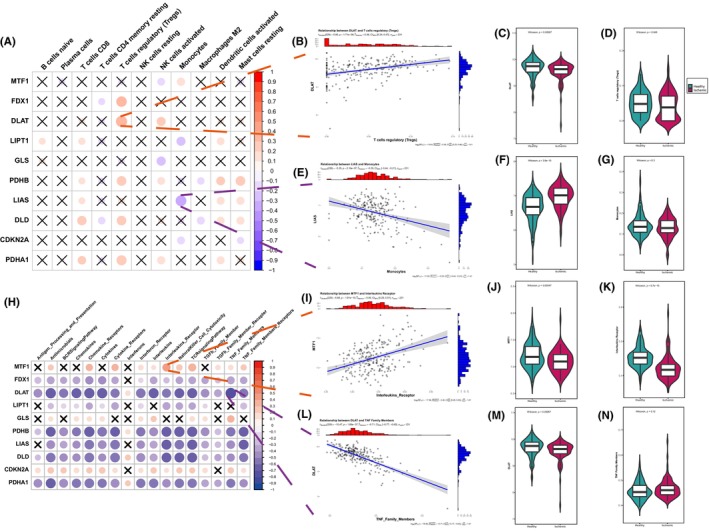
The correlation between infiltrating immunocytes, immune reaction gene‐sets and cuproptosis genes. (A) The dot plot demonstrated the correlations between each dysregulated immune microenvironment infiltration cell type and each dysregulated cuproptosis gene. (B) The most positive correlated immunocyte‐cuproptosis gene pair is DLAT‐T cells regulatory (Tregs). (C, D) The violin plot of DLAT expression and Tregs infiltration in IHF. (E) The most negative correlated immunocyte‐cuproptosis pair is LIAS‐monocytes. (F, G) The violin plot of LIAS expression and monocytes infiltration in IHF. (H) The dot plot demonstrated the correlations between each dysregulated immune reaction gene‐sets and each dysregulated cuproptosis gene. (I) The most positive correlated pair is MTF1‐Interleukins_Receptor. (J, K) The violin plot of MTF1 expression and Interleukins_Recepor reaction in IHF. (L) The most negative correlated pair is DLAT‐TNF_Family_Members. (M, N) The violin plot of DLAT expression and TNF_Family_Members reaction in IHF. Wilcoxon rank‐sum test was employed for significant testing of each dysregulated cuproptosis gene and immune microenvironment infiltration cell type or immune reaction gene‐sets.

The relationship between cuproptosis genes and infiltrating immunocytes were demonstrated in Figure 3A. Dysregulated cuproptosis genes were observed closely linked to various infiltrating immunocytes in the microenvironment. In particular, a significant positive correlation was found between regulatory T cells (Tregs) and several genes key to the cuproptosis regulatory pathway, including FDX1, DLD and three PDH complex genes (DLAT, PDHB and PDHA1). This implies a connection between the increased infiltration of Tregs and the gene expression of multiple cuproptosis‐related pathways in IHF, especially that of DLAT (Figure 3B–D). Additionally, monocytes showed the most pronounced negative correlation with LIAS expression (Figure 3E–G).

Correspondingly, the correlation between cuproptosis genes and immune reaction gene‐sets were presented in Figure 3H. Most of these gene sets displayed a substantial negative correlation with several dysregulated cuproptosis genes in IHF samples, such as FDX1, DLD, LIPT1, LIAS, DLAT, PDHB and PDHA1. Notably, DLAT was most negatively correlated with the TNF family member receptor (Figure 3L–N), while MTF1 displayed the strongest positive correlation with interleukins receptor (Figure 3I–K). These findings highlight the significant role of cuproptosis genes in IHF in relation to a range of immune processes.

### 3.4 Two cuproptosis expression patterns in IHF

**FIGURE 4:**
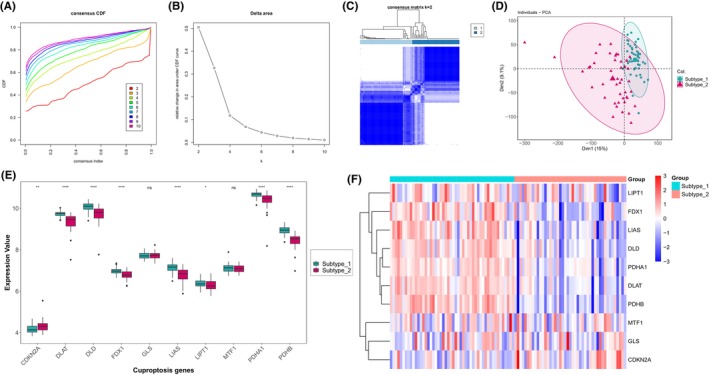
Unsupervised clustering of 10 cuproptosis genes identifying 2 distinct cuproptosis‐mediated regulation pattern subtypes in IHF. (A) Consensus clustering cumulative distribution function (CDF) for *k* = 2–10. (B) Relative change in area under the CDF curve for *k* = 2–10. (C) Heatmap of the matrix of co‐occurrence proportions for IHF samples. (D) Principal component analysis (PCA) for the transcriptome profiles of 2 cuproptosis regulation patterns, demonstrating a clear distinction in transcriptome between different regulation patterns. (E, F) The box‐plot and heatmap‐plot demonstrate the expression pattern of 23 significantly dysregulated cuproptosis genes between 2 cuproptosis regulation patterns. Wilcoxon rank‐sum test was employed for significant testing.

To investigate the expression patterns of cuproptosis in IHF, we performed an unsupervised consensus clustering analysis on IHF samples, utilizing the expression data of 10 cuproptosis genes (Figure 4A–C). As a result, we identified two distinct IHF sample subtypes based on their cuproptosis expression patterns. Subtype‐1 consisted of 50 samples, while subtype‐2 contained 45 samples (Figure 4D). We then conducted a comparative analysis of the expression of the 10 cuproptosis genes across these two subtypes. This comparison revealed significant variations in the expression of eight cuproptosis genes between the subtypes (Figure 4E,F). Specifically, subtype‐1 demonstrated elevated expression levels in a majority of the cuproptosis genes, including FDX1, DLD, LIAS, LIPT1, DLAT, PDHA1 and PDHB. Conversely, a higher expression level of CDKN2A was observed in subtype‐2. These findings suggest that these eight cuproptosis genes might be implicated in distinct regulatory mechanisms influencing the progression of IHF.

### 3.5 Immune microenvironment characteristics in distinct cuproptosis expression patterns

**FIGURE 5:**
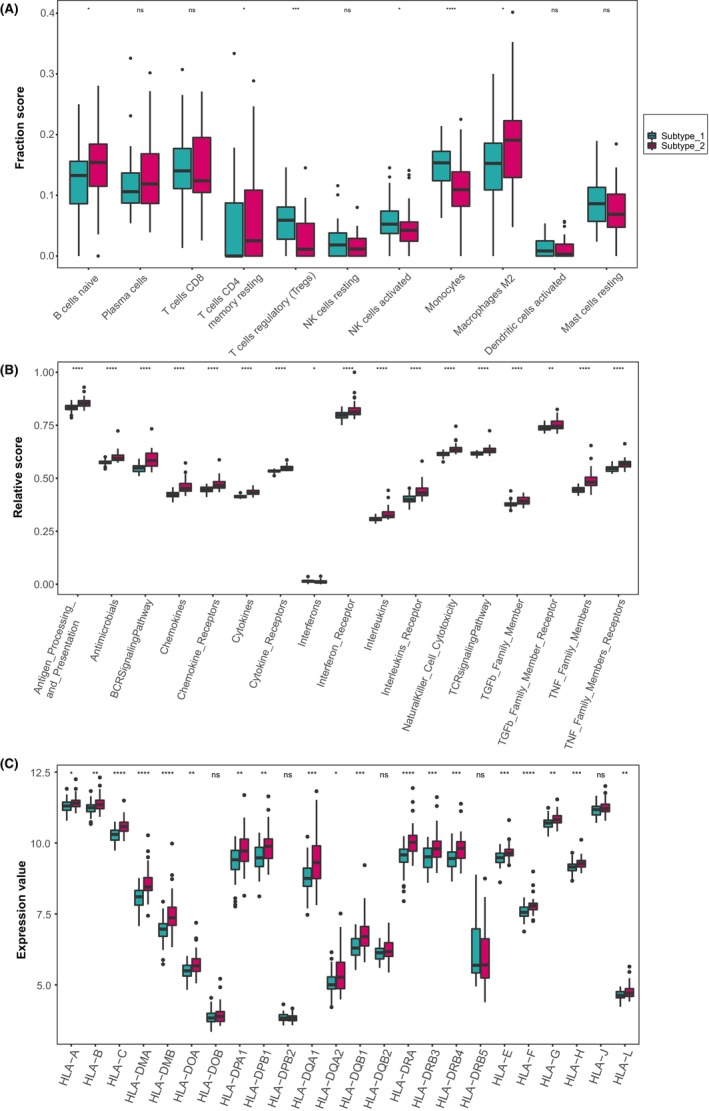
Diversity of immune microenvironment characteristics between distinct cuproptosis mediated regulation patterns. (A) The abundance differences of infiltrating immunocytes between 2 cuproptosis mediated regulation patterns. (B) The activity differences of immune reaction gene‐sets between 2 cuproptosis mediated regulation patterns. (C) The expression differences of HLA genes between 2 cuproptosis mediated regulation patterns. Wilcoxon rank‐sum test was employed for significant testing.

To delve deeper into the differences in immune microenvironmental characteristics between the two identified IHF subtypes, we assessed the abundance of infiltrating immune cells, the activity of immune response gene‐sets and the expression of HLA genes as key indicators. As Figure 5A demonstrated, subtype‐1 exhibited relatively higher levels of infiltrating Tregs, resting NK cells, activated NK cells and monocytes. In contrast, subtype‐2 was enriched in naive B cells, plasma cells, CD8+ memory T cells, CD4+ memory resting T cells and M2 macrophages. As for the immune response gene‐sets, subtype‐2 displayed higher levels in most chemokines, cytokines and signalling pathways, while subtype‐1 had a higher level in interferons (Figure 5B). Likewise, most HLA genes displayed higher expression levels in subtype‐2 (Figure 5C). These results suggested that subtype‐1 was likely to be associated with innate immunity and immune regulation, whereas subtype‐2 was primarily associated with adaptive immunity, indicating multiple immune processes were probably related with the expression of cuproptosis genes.

### 3.6 Biological functions behind cuproptosis expression patterns

**FIGURE 6:**
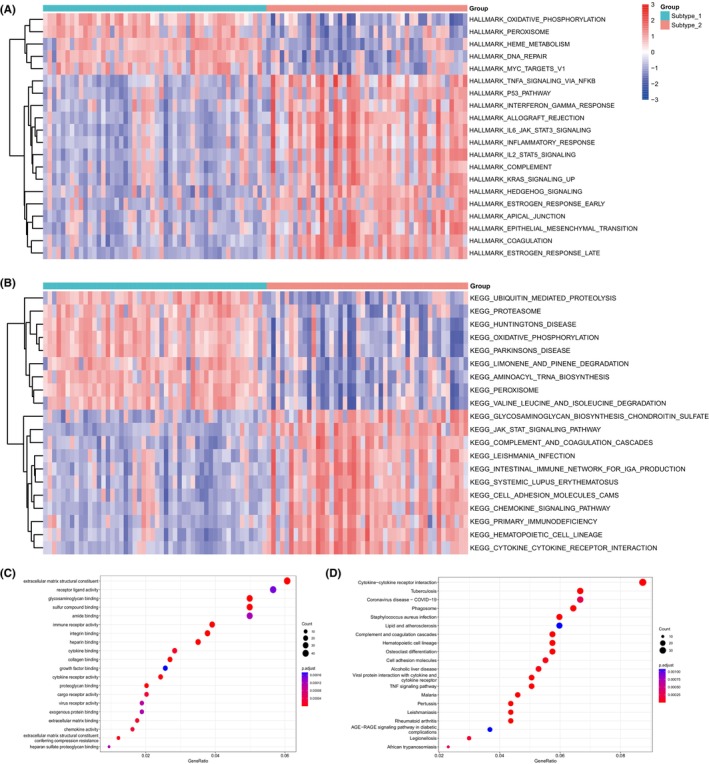
The underlying biological characteristics diversity between 2 cuproptosis mediated regulation patterns. The top 20 HALLMARKS (A) and KEGG (B) pathways with the most significant differences between 2 cuproptosis mediated regulation patterns. GO‐BP functional (C) and KEGG enrichment (D) analysis for cuproptosis related genes.

The distinct expression patterns of cuproptosis genes and the characteristics of the immune microenvironment in IHF point to two primary expression profiles and their respective regulatory mechanisms. To gain a deeper understanding of the respective roles of the two subtypes and identify cuproptosis‐related biological signalling pathways, a comparative analysis was conducted in the HALLMARKS pathway and KEGG pathway. GSVA enrichment analysis was performed to calculate the enrichment scores. As illustrated in Figure 6A,B, subtype‐1 demonstrated a higher enrichment in biological processes and structures related to energy metabolism, such as oxidative phosphorylation and peroxisome. In contrast, subtype‐2 appeared to be more involved in inflammation related processes, like the classic JAK/STAT signalling pathway. Furthermore, subtype‐2 exhibited notable associations with various inflammatory chemokines, cytokines and cytokine‐cytokine receptor interactions. Some inflammatory cytokines were found enriched in subtype‐2, including the TNF‐α and IL‐6.

The GO‐BP enrichment analysis revealed significant involvement of extracellular matrix structural constituents, receptor ligand activity, glycosaminoglycan binding, sulphur compound binding and amide binding, as shown in Figure 6C. Moreover, the KEGG enrichment results emphasized the strong relevance of cytokine‐cytokine receptor interaction, as depicted in Figure 6D. This analysis also revealed the relevance of several immune processes such as the complement and coagulation cascades, cell adhesion molecules, cytokine receptor and TNF signalling pathway. Additionally, the analysis linked cuproptosis with certain infectious diseases, including tuberculosis, COVID‐19 and staphylococcus aureus infection, suggesting a potential role of cuproptosis in the immune response to these infections. These insights are consistent with the results obtained from the GSVA analysis.

### 3.7 Co‐expression network analysis identified cuproptosis mediated regulation pattern related gene modules

**FIGURE 7:**
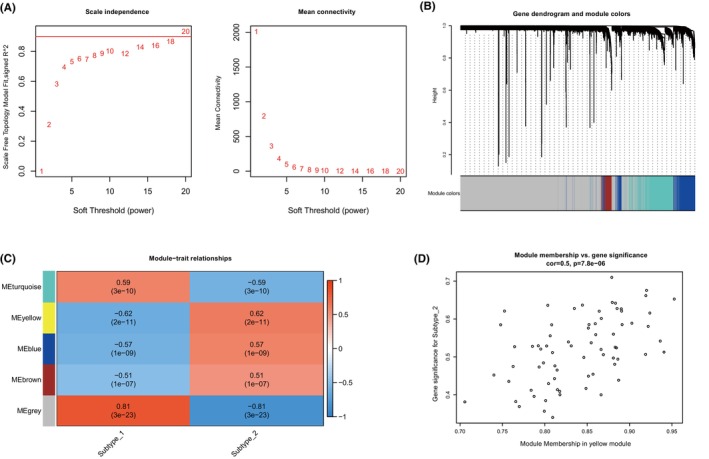
Co‐expression gene modules related to cuproptosis mediated regualtion patterns. (A) Analysis of the scale‐free fit index and analysis of the mean connectivity for various soft‐thresholding powers. (B) Gene dendrogram obtained by average linkage hierarchical clustering. The colour row underneath the dendrogram shows the module assignment determined by the Dynamic Tree Cut, in which 5 modules were identified. (C) Heatmap of the correlation between module eigengenes and the cuproptosis mediated regulation subtypes. (D) A scatterplot of gene significance (GS) for cuproptosis mediated subtype‐2 versus module membership (MM) in the yellow module.

Subsequently, we utilized Weighted Gene Co‐expression Network Analysis (WGCNA) to create a comprehensive gene network that correlates with the expression patterns of cuproptosis. This approach enabled us to identify gene co‐expression modules that are linked to distinct cuproptosis regulatory mechanisms, as depicted in Figure 7A,B. We successfully identified five gene modules and allocated the corresponding genes to each specific pattern, as shown in Figure 7C. Notably, among the five identified modules, the yellow module exhibited the strongest association with the cuproptosis subtype as demonstrated in Figure 7D. This module showed a particularly notable correlation with subtype‐2, characterized by a correlation coefficient of 0.5 and a highly significant *p*‐value of 7.8e‐6. These findings reveal a complex network of gene expression regulation influenced by cuproptosis, providing deeper insights into its role in the molecular landscape.

## 4 DISCUSSION

Cuproptosis is a newly identified programmed cell death pattern characterized by intracellular copper overload and protein lipidation. Since research on the molecular mechanisms of cuproptosis is limited, our analysis focused on the 10 relevant proteins and genes reported in the original study.^12^ Our study utilized the GSE57338 dataset, which comprised 136 healthy samples and 95 ischemic samples. Our analysis of these samples revealed notable changes in the expression of genes associated with the PDH complex. DLAT and MTF1 showed significant downregulation in expression, whereas PDHB and GLS showed upregulation. Additionally, genes related to the LA pathway, namely LIAS, LIPT1 and DLD, showed a uniform increase in expression levels. The inconsistency between the expression levels of LA pathway genes and PDH complex genes may indicate PDH complex protein oligomerization, underscoring the involvement of cuproptosis in IHF. Regarding FDX1, a significant decrease in expression was observed. FDX1 is considered as a key regulator in cuproptosis,^12^ not only regulates PDH lipoylation, but also reduces Cu^2+^ to Cu^+^, thereby intensifying cellular copper toxicity. Moreover, FDX1 also participates in the formation of Fe‐S clusters.^41^ A reduction in the stability of Fe‐S clusters was also mentioned in the original study on cuproptosis.^12^ The observed reduction in FDX1 expression might represent a myocardial cellular defence mechanism against cuproptosis. Similar patterns have been identified in tumour tissues, suggesting that such changes could aid tumour evasion of cuproptosis and facilitate tumour growth.^42^, ^43^, ^44^ This raises the possibility of compensatory regulatory mechanisms within the body that protect cells from copper toxicity by downregulating FDX1 expression during the prolonged progression of heart failure post‐ischemic events. This area merits further investigation to unravel the intricate mechanisms involved.

Our investigation into the protein–protein interactions and gene expression correlations related to cuproptosis in these samples revealed a significant association between the LA pathways and the PDH complex genes. Although as shown in Figure 1D, DLD exhibited the highest correlation with PDHB across all samples, and with PDHA1 specifically in ischemic samples. In fact, the three genes of the LA pathways (DLD, LIAS and LIPT1) demonstrated a strong positive correlation with three other PDH complex genes (DLAT, PDHA1 and PDHB). This suggests a critical role of protein lipoylation in IHF tissue. Considering the previously observed discordance in gene expression between PDH complex‐related genes and LA pathway‐related genes, it suggests that ischemic events may have already exerted a substantial impact on the TCA cycle and energy metabolism of cardiomyocytes. The observed significant cuproptosis damage during ischemic events and the consequent downregulation of FDX1 expression, along with the upregulation of LA pathway‐related gene expression, likely represent compensatory changes in response to ischemic conditions. Hence, similar to common indicators of heart failure severity such as LVEF and BNPs, the expression of cuproptosis genes could serve as an indicator to assess the severity of heart failure and its compensatory changes. Building upon the identification of differential expression of cuproptosis genes in IHF left ventricles, we screened 10 cuproptosis genes using a series of bioinformatics methods and developed a risk diagnosis model capable of effectively distinguishing healthy and IHF samples. This cuproptosis IHF prediction model achieved a notable AUC of 0.95 for ROC curve, effectively distinguishing between healthy individuals and those with IHF. Furthermore, its robustness was confirmed through external validation, where the model demonstrated an AUC of 0.72 by analysing novel IHF samples from three additional GSE databases. These results underscore the model's reliability and potential for broader application. We are optimistic that this preliminary bioinformatics study will lay the groundwork for future clinical applications, especially in the prediction of heart failure risk.

We further investigated the correlations between cuproptosis genes and immune characteristics, focusing on infiltrating immunocytes and immune reaction gene‐sets. The cuproptosis genes did not exhibit broad correlations with infiltrating immune cells, with the exception of a notable correlation with Tregs. There was a strong positive correlation observed between Tregs and several genes involved in the lipoylation of the pyruvate dehydrogenase (PDH) complex, specifically FDX1, DLD, DLAT, PDHA1 and PDHB. This suggests that Tregs are significantly associated with multiple genes within this pathway. Prior research has indicated that Tregs are recruited to the myocardium to regulate excessive inflammation and mitigate cardiac deterioration in the initial days after myocardial infarction, a period during which cuproptosis could occur extensively.^45^ In the remodelling phase of chronic IHF, the infiltration of Tregs promotes wound healing by influencing monocyte trafficking and macrophage M2 differentiation.^46^ The correlation between Tregs and cuproptosis genes indicates that Treg‐regulated immune reactions are extensively involved in the compensatory regulation processes of the heart following ischemic events and cuproptosis.

As for immune reaction gene‐sets, we observed a significant negative correlation between the majority of cuproptosis genes and most immune reaction gene‐sets. This aligns with other studies exploring the interplay between cuproptosis and immune responses in other diseases.^47^, ^48^ Utilizing these immune characteristics, we categorized the IHF sample expression patterns influenced by cuproptosis into two subtypes. From an immune feature perspective, subtype‐1 is predominantly associated with innate immunity and immune regulation, whereas subtype‐2 is primarily associated with adaptive immunity. Specifically, immune response gene‐sets and HLA gene expression are significantly enriched in subtype‐2. Further analysis through HALLMARKS and KEGG pathways revealed the presence of multiple inflammatory pathways in subtype‐2, such as the classic inflammatory pathways like the TNF‐α/NF‐κB pathway and the IL‐6/JAK/STAT3 pathway. Both TNF‐α and IL‐6, known as pro‐inflammatory cytokines, are present during acute and chronic phases post‐ischemic events. TNF‐α is associated with impaired systolic and diastolic function, as well as adverse cardiac remodelling in chronic heart failure.^49^ IL‐6 possesses both pro‐inflammatory and anti‐inflammatory functions. Although its ultimate negative inotropic effect is detrimental to ventricular remodelling,^50^ it promotes recovery after MI by facilitating processes such as angiogenesis and haematopoiesis.^51^ These insights reinforce the notion of concurrent cell death and repair processes in IHF, highlighting the need for further research to understand these complex underlying mechanisms.

In summary, our findings uncovered the cuproptosis‐mediated regulation patterns in IHF, providing insights into the impact of cuproptosis on the immune microenvironment in IHF. This study was the first systematic investigation of the potential links between cuproptosis and the immune microenvironment in IHF. Our findings may provide valuable insights for future mechanistic research on the relationship between cuproptosis and IHF.

## AUTHOR CONTRIBUTIONS

**Zhebin Chen:** Conceptualization (equal); formal analysis (lead); writing – original draft (lead). **Yunhui Zhu:** Conceptualization (supporting); formal analysis (equal); writing – original draft (equal). **Songzan Chen:** Investigation (equal); writing – original draft (supporting). **Zhengwei Li:** Investigation (equal). **Guosheng Fu:** Writing – review and editing (equal). **Yao Wang:** Conceptualization (lead); formal analysis (equal); writing – review and editing (lead).

## FUNDING INFORMATION

This research received no external funding.

## CONFLICT OF INTEREST STATEMENT

The authors declare that they have no conflict of interest.

## Supporting information


Figures S1–S3.

## Data Availability

The data that support the findings of this study are available in [https://www.ncbi.nlm.nih.gov/geo/query/acc.cgi?acc=GSE57338]. All the raw codes are provided as a part of Supplementary Materials and are available from the corresponding author.
