# Supplementary material for: Immune patterns of cuproptosis in ischemic heart failure: A transcriptome analysis
Source: J Cell Mol Med. 2024 Mar 20;28(7):e18187. doi: 10.1111/jcmm.18187 (PMC10955177; doi:10.1111/jcmm.18187)
Supplement: Supplementary file 1 — Figures S1–S3. [file JCMM-28-e18187-s001.docx]

**Supplementary Materials**

**Immune Patterns of Cuproptosis in Ischemic Heart Failure: A Transcriptome Analysis**

Zhebin Chen M.D.*, Yunhui Zhu M.D.*, Songzan Chen M.D., Zhengwei Li M.D., Guosheng Fu M.D. Ph.D., Yao Wang M.D.^#^

Department of Cardiology, Key Laboratory of Cardiovascular Intervention and Regenerative Medicine of Zhejiang Province, Sir Run Run Shaw Hospital, School of Medicine, Zhejiang University, Hangzhou 310016, People’s Republic of China.

# Yao Wang M.D. is the corresponding author of this article.

* Zhebin Chen and Yunhui Zhu contributed equally to this article.

Correspondence to Yao Wang M.D.: Department of Cardiology, Key Laboratory of Cardiovascular Intervention and Regenerative Medicine of Zhejiang Province, Sir Run Run Shaw Hospital, School of Medicine Zhejiang University, 3 East Qingchun Road, Hangzhou, Zhejiang Province, People’s Republic of China;

Telephone number: +86 0571-86006246;

Fax number: +86 0571-86006246;

E-mail: [11618395@zju.edu.cn](mailto:11618395@zju.edu.cn)


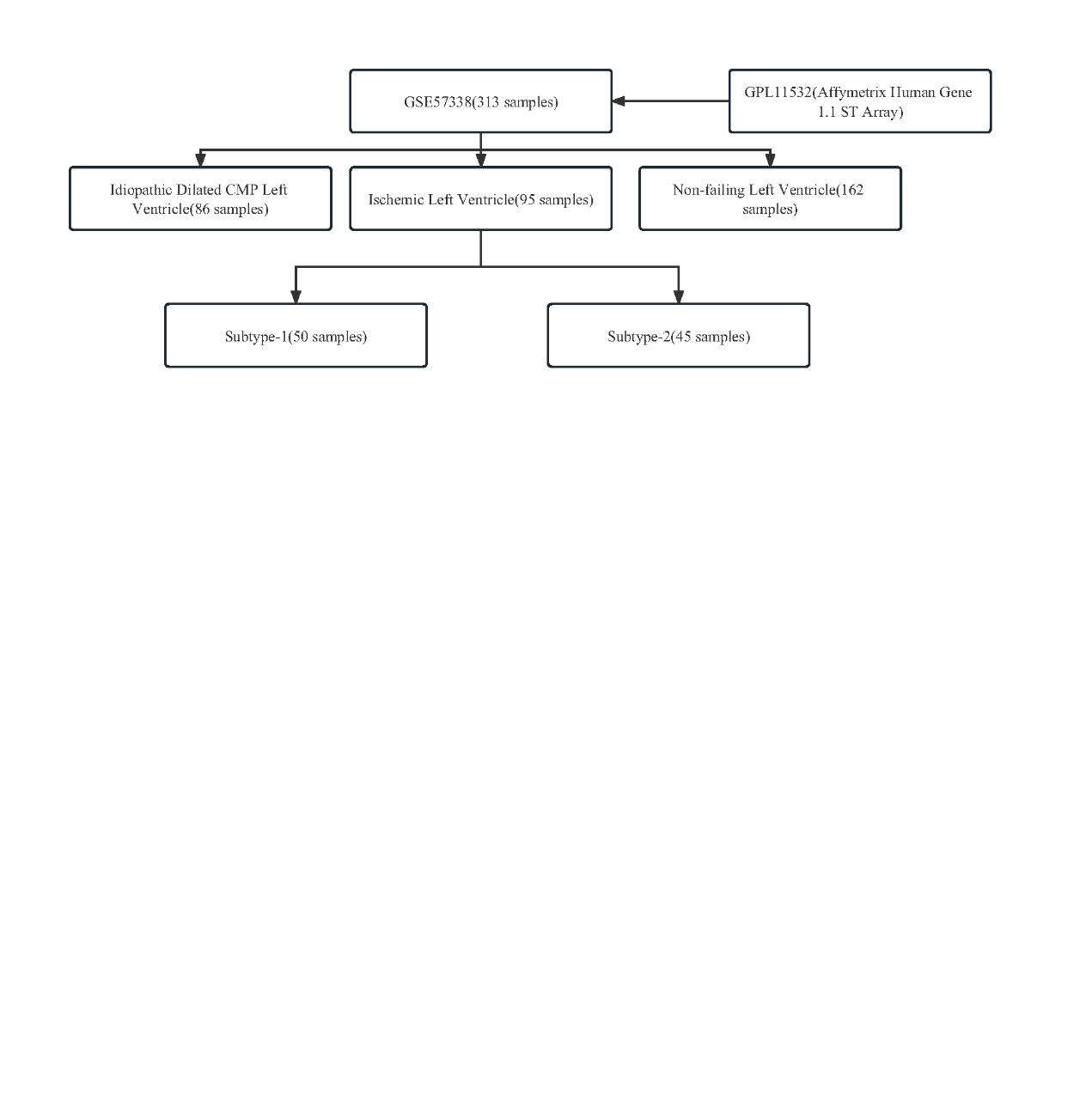


**Figure 1. Flowchart.**

**
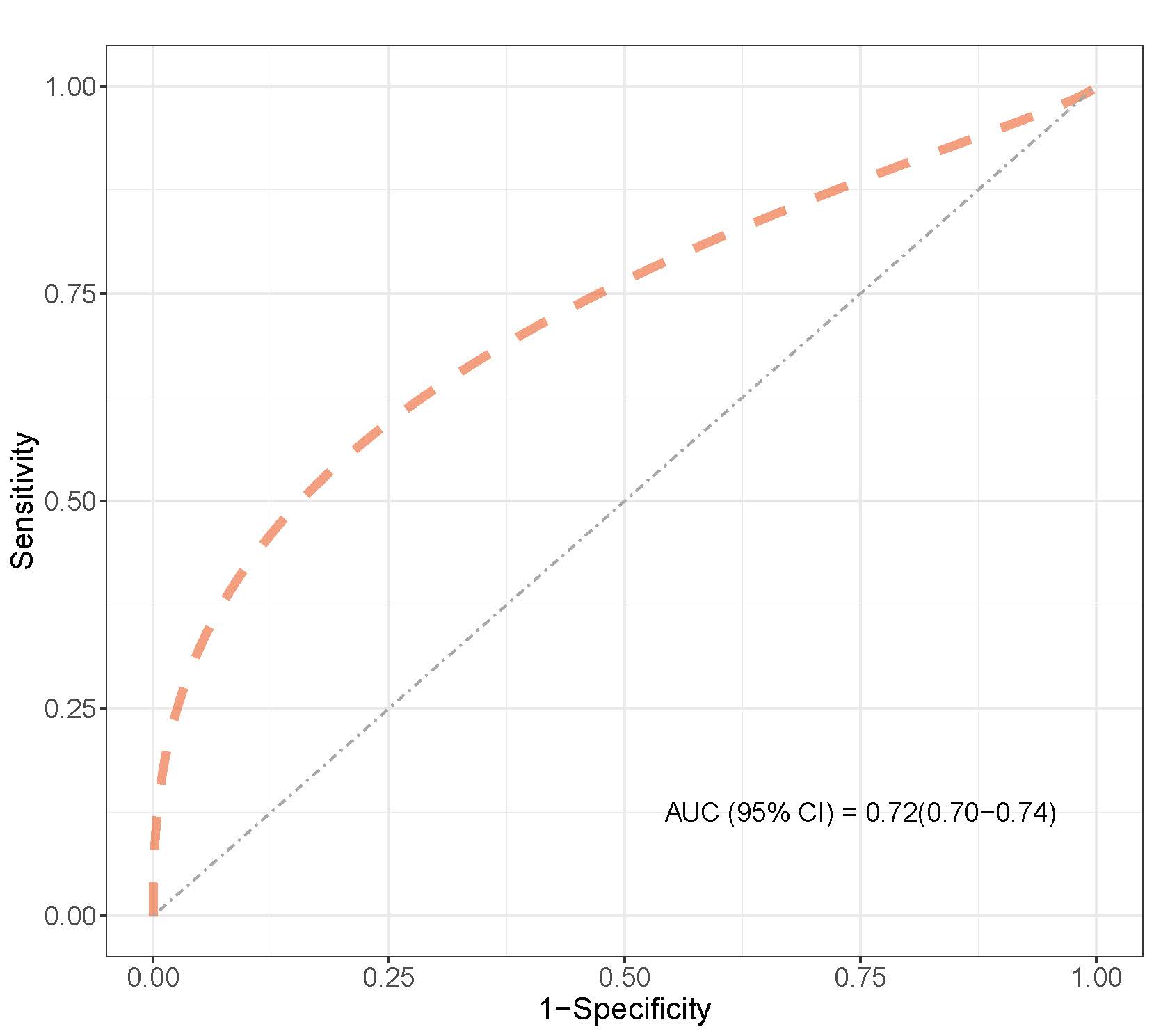
**

**Figure S2. ROC curve with AUC value for robustness validation of the cuproptosis IHF prediction model.** Three additional databases GSE26887 (5 healthy samples and 12 IHF samples), GSE42955 (5 healthy samples and 12 IHF samples), and GSE76701 (4 healthy samples and 4 IHF samples) were utilized for validation.

**
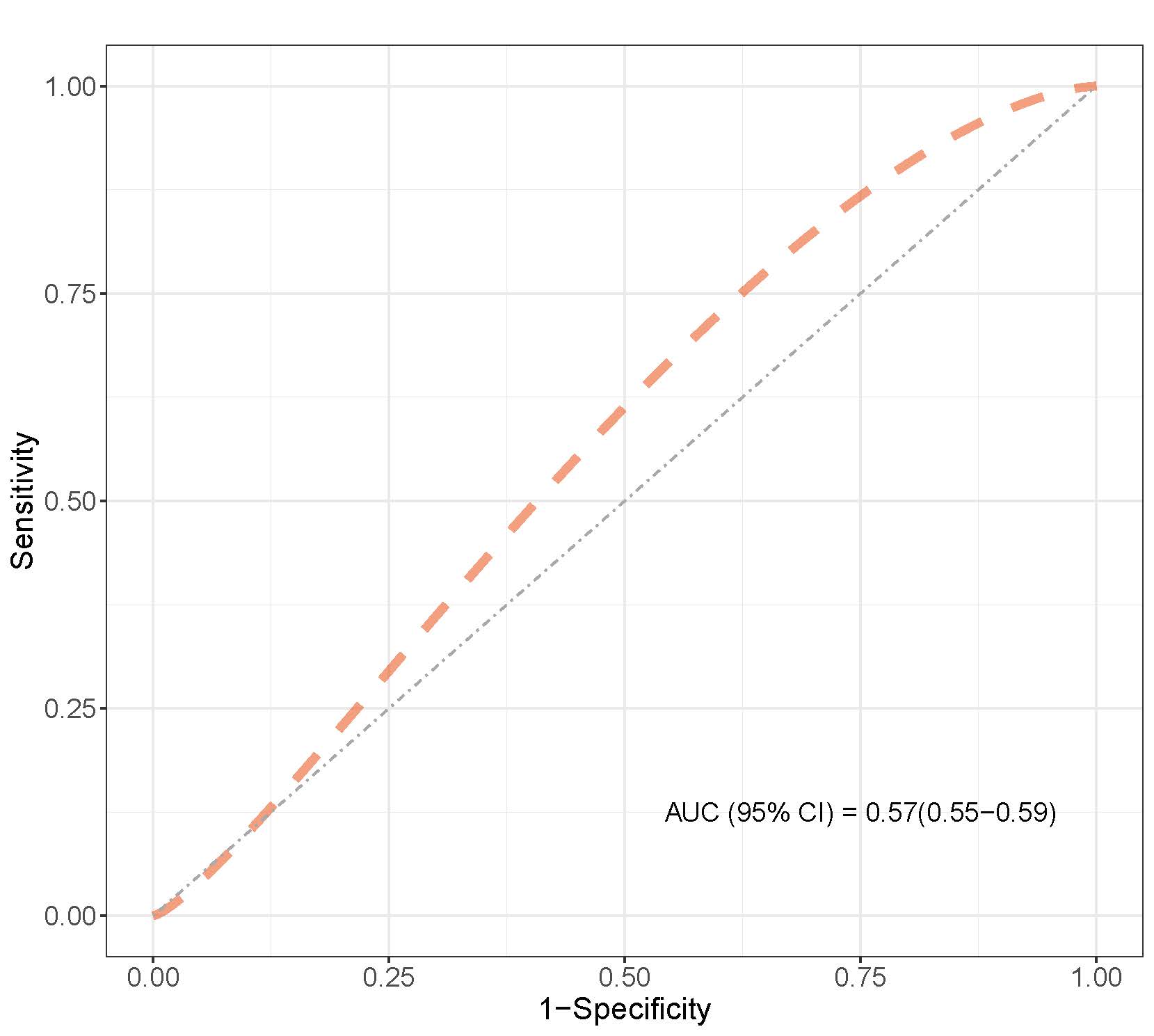
**

**Figure S3. ROC curve with AUC value for the discrimination ability of IHF prediction model for distinguish two cuproptosis expression patterns in IHF samples.**
